# Age sequences of the elderly’ social network and its efficacies on well-being: an urban-rural comparison in China

**DOI:** 10.1186/s12877-020-01773-8

**Published:** 2020-09-29

**Authors:** Zhenhua Zheng, Hong Chen

**Affiliations:** 1 College of Communication and Art Design, University of Shanghai for Science and Technology, No.516, Jungong Road, Shanghai, 200093 China; 2 College of Architecture & Environment, Sichuan University, No.24 First South Section First Ring Road, Chengdu, 610065 China

**Keywords:** Social network, Age sequence, Family network, Friend network, Well-being

## Abstract

**Background:**

Although social network is a known determinant of the elderly’s well-being, it is not clear, in urban-rural and age-comparison, what its structural characteristics are and how it works for well-being. The research aims to discuss the features of the elderly’s social network and the social network efficacies on the well-being of older adults in China’s urban and rural areas as well as revealing the urban-rural disparities among the elderly of different age groups.

**Methods:**

In this study, descriptive statistical analysis and structural equation Modeling (SEM) were used to make a group comparison between the urban and rural elderly of different age groups. All data are quoted from 2014 China Longitudinal Aging Social Survey (CLASS). The survey adopted the multi-stage probability sampling method, targeting Chinese senior citizens aged 60 and above, the ultimate samples totaled 11,511.

**Results:**

The social network of the elderly in China feature a “reverse structure” in age sequences: with ageing, family network of the elderly expand while their friend network shrink; also, the expansion scale of the rural elderly’s family network is significantly larger than that of the city’s while the shrinkage scale of their friend network is smaller compared with its urban counterpart. The effect of family network on the rural elderly’s well-being shows a remarkable increase with age. However, there is no noticeable change in urban elderly groups of different ages.

**Conclusion:**

The social network characteristics of the Chinese elderly are different between different age stages. Namely, the family network and the friend network have the “reverse structure “ in age sequences. Meanwhile, the family network and the friend network have different efficacies on the well-being of the elderly in China, and the differences between urban and rural areas are even more obvious. For rural elderly, family network has very important effects on their well-being. Moreover, With the increase of age, family network’s efficacies increase gradually. For urban elderly, comparatively, family network is just as important as friend network.

## Background

The socializing and social network of older adults have long been one of the major concerns of the international community [1, 2]. With the changes of their social roles and the decline in physiological functions, the elderly are often confronted with the problems of negative feelings and illnesses. Compared with young people, they need more support from social network to meet their physical, social, and emotional needs [3, 4].

Existing research on older adults’ social network mainly involves two aspects: characteristics and efficacies on well-being. Research on characteristics reveals two different points of views. One is that senior citizens’ social network scale is relatively small and will be smaller with aging [5–7]. The other claims that although their friend network may narrow down, their family network tend to expand [3, 8]. So far, no agreement has been reached regarding social network’s efficacies on older adults’ well-being. Most scholars are inclined to agree that social network can greatly promote the well-being of the elderly groups [9–13]. However, some scholars have obtained different conclusions. Fuller-Iglesias & Antonucci (2016) [14] pointed out that the scale of social network doesn’t significantly improve the life satisfaction of the elderly. The impact of social network on the well-being of the elderly depends on the specific social support it can provide [15, 16] or on the network structure and the quality of interpersonal relationships [17–19].

Based on the summary of the existing research, there is no consist conclusion on the characteristics of the elderly’s social network or the efficacies of the social network on their well-being. This is due to three reasons: the structural complexity of social network, the heterogeneity of groups of older adults, and the different sources of data. First, for elderly people, their social network are established mainly in two dimensions: family and friend [20, 21], which have remarkable differences in terms of ways of communication, emotional connotations, mechanisms of efficacies on well-being, etc. [8, 22]. Some scholars have pointed out that family network have a stronger effect on the well-being of the elderly [23], but others have found that friend network play a greater role under certain circumstances [17, 24]. Therefore, deviations in research conclusions will be inevitably produced if the social network is considered as a whole research objective. Second, due to the noticeable heterogeneity in different elderly groups, the physical and psychological conditions of elderly people at different ages would vary, ignoring the age differences in research may cause deviations in information and conclusion [25–28]. In addition, the characteristics of social network formed by different social backgrounds and cultural traditions are different. Therefore, data of the sample from different regions also could lead to differences in research results [29, 30].

This study focused on the elderly in China. Though China’s economy is getting tightly connected with the world, it has unique in terms of pension models, family relationships, and cultural background. As Chinese culture is rooted in Confucian values, Chinese families are more like a “community” [31]. The elderly in China are highly dependent on their family members, especially on their children. In addition, the elderly samples of China also have differences between urban and rural area [32, 33]. On the one hand, the economic development of Chinese cities is significantly faster than that of rural areas. The income level and quality of life of urban elderly are also higher than those of rural elderly [34]. On the other hand, there are differences in the implementation of the family planning system in urban and rural areas. The family planning policy is strictly implemented in cities, and a couple can only have one child. However, in many rural areas, the one-and-a-half child policy has long been adopted, which means that if the first child of a couple is a girl, they can have a second child. Therefore, in China, a country with distinctive characteristics of urban-rural dual structure, there must be differences in the structural characteristics of the elderly’s social network, as well as in the mechanisms of their efficacies on well-being of the urban and rural areas. However, the existing literatures lack comparative studies in terms of different dimensions of the social network, different age stages of the elderly, and the difference between the Chinese urban and rural areas.

Our study compared multiple groups of elderly people in China. The research objectives include the following aspects: (1) Through the comparison among different age stages, reveal the characteristics of the social network of the elderly in China urban and rural areas; (2) To compare the efficacies of social network in different dimensions on the well-being of the elderly in China; (3) To compare the efficacies of social network on the well-being of urban-rural elderly in different age stages. Our study is expected to: theoretically enrich the research content on the characteristics of the elderly’s social network and its efficacies on well-being with the research conclusions from Chinese samples. Moreover, in respect of practice, our conclusions provide a reference for the formulation of public policy for the elderly in China and help promote the development of active ageing.

## Methods

### Study population

All data in this article are quoted from 2014 China Longitudinal Aging Social Survey (CLASS). The survey was conducted by Chinese Investigation and Statistics Center of Renmin University of China from June 1 to August 31, 2014. The Academic Committee of the School of Statistics of Renmin University of China is responsible for the ethical approval of the survey data and related issues. It aims to collect the data regularly and systematically about the socio-economic backgrounds and the situation of children of Chinese elderly in order to find out a variety of problems that they have to face when aging. It is thus expected to provide statistical basis for the study of aging in China and the solution to this issue. The CLASS (2014) survey adopted the multi-stage probability sampling method. The Primary Sampling Units (PSU) were selected at county-level areas, including counties, county-level cities, and districts, and village / neighborhood committees were selected as the Secondary Sampling Units (SSU). Map sampling was used in each SSU to obtain survey samples. The survey targets are Chinese citizens aged 60 and above (no age limit) living in the current address. The survey was conducted in the form of face-to-face interviews and reading questionnaires. That is, the interviewer reads the questions and answers one by one according to the questionnaire, the interviewee selects the corresponding answer item, and then the interviewer records them on the questionnaire. The survey covers 29 provinces, autonomous regions, municipalities in China. The final sample includes 134 counties, districts, and 462 villages with a total of 11,511 people.

Internationally those of 60 or 65 are generally regarded as senior citizens. In China, the academic community tends to agree that the elderly can be divided into three tiers based on their actual age [35, 36], that is, the young-old (60–69), the middle-old (70–79), and the old-old (80+). We therefore divided our subjects accordingly into these three categories. The valid urban samples turned out to include 3565 young-old subjects, 2200 middle-old subjects, and 1142 old-old subjects. For the valid rural samples, the numbers were respectively 2451, 1440, and 713.

### Measurement of social network

Social network refers to a collection of specific individuals and the various relationships that connect them (such as friendship, communication, and the offer of advice) [37]. For the elderly, the intimate relationship with families and friends count more as their working and social functions decline [3]. Regarding social network measurement, Lubben Social Network Scale 6 (LSNS-6) designed by Lubben et al. (2006) [21] has been widely acknowledged [8, 38]. CLASS also used this scale in its survey to collect the data of older adults’ social network. The scale includes 6 indicators in two categories of family network and friend network. The former includes 3 indicators of family contacts, family talks, and family supports, and the latter comprises 3 indicators of friend contacts, friend talks, and friend supports. The description of each variable measurement is shown in Table 1.

**Table 1:** Measurement of social network and older adults’ well-being

Latent variables		Observed variables	Problems	Values
Independent variable: Social network	Family network	Family contacts	How many family members/relatives do you see or hear from at least once a month?	0 = none,1 = 1,2 = 2,3 = 3–4,4 = 5–8,5 = 9 and above
		Family talks	How many family members/relatives do you feel at ease with that you can talk about private matters?	0 = none,1 = 1,2 = 2,3 = 3–4,4 = 5–8,5 = 9 and above
		Family supports	How many family members/relatives do you feel close to such that you could call on them for help?	0 = none,1 = 1,2 = 2,3 = 3–4,4 = 5–8,5 = 9 and above
	Friend network	Friend contacts	How many friends do you see or hear from at least once a month?	0 = none,1 = 1,2 = 2,3 = 3–4,4 = 5–8,5 = 9 and above
		Friend talks	How many family members/relatives do you feel at ease with that you can talk about private matters?	0 = none,1 = 1,2 = 2,3 = 3–4,4 = 5–8,5 = 9 and above
		Friend supports	How many family members/relatives do you feel close to such that you could call on them for help?	0 = none,1 = 1,2 = 2,3 = 3–4,4 = 5–8,5 = 9 and above
Dependent variables: older adults’ well-being		life satisfaction	Overall, are you satisfied with your current life?	1 = very unsatisfied, 2 = quite unsatisfied, 3 = average, 4 = quite satisfied, 5 = very satisfied
		Self-rated health	How do you think of your current health condition?	1 = very unhealthy, 2 = quite unhealthy, 3 = average, 4 = quite healthy, 5 = very healthy

### Measurement of well-being

Well-being takes place in the realm of human psychology and spirit, which is a sense of happiness and benefits obtained via subjective judgment and feelings [39]. In early studies of psychology and sociology, well-being was a general concept based on the measurement of 3 indicators – life satisfaction, positive feelings, negative feelings [40]. In later pertinent studies, indicators used by researchers in the measurement of well-being have varied to some extent. For example, Nguyen et al. (2016) [23] refer to the three indicators of life satisfaction, happiness, and self-esteem; Fuller-Iglesias & Antonucci (2016) [14] used life satisfaction, self-rated health, depressive symptoms and chronic health conditions in measurement. No matter which indicators researchers may turn to, what they have in common is that the indicators are centered on life satisfaction and subjective judgment, in which life satisfaction has remained a core measuring variable as it can best manifest individual life quality. However, for the elderly in particular, the significance of health has far surpassed everything else. The self-rated health of the elderly not only includes the comprehensive evaluation of their physical health, but also reflects the information on their psychological status [41]. Self-rated health of the elderly is a more comprehensive psychological measurement index [42]. Therefore, this research refers to life satisfaction and self-rated health as measuring indicators of older adults’ well-being.

### Control variables

This article included age, gender, number of children, marital status, income and education into the model as control variables. Gender and marital status are two-category variables, and marital status is divided into two categories: spouse and non-spouse. Income measures the total sum of the personal income of older adults in the past year and in this article income is measured on 5 tiers. This is because income data often are rough numbers when they are obtained, and what’s between income and well-being is not a simple linear relationship. Therefore, it is not meaningful to study how much health is improved with the increase of certain amount of income. Only by comparing well-being of different income groups can it better promote policy-making and ensure its implementation [43]. That explains why most sociologists would stratify income data in their research [44, 45]. The values of older adults’ income level in this article are classified according to the 20, 40, 60 and 80% quantiles of the total sample income value of the CLASS data. The final values are ranked from low to high as: 2000 Chinese Yuan (CHY) and below is low-income level with the value of 1; 2001–7200 CHY is lower-middle income level with the value of 2; 7201–20,000 CHY is middle-income level with the value of 3; 20,001–32,164 CHY is upper-middle income level with the value of 4; 32,164 CHY and above is high-income level with the value of 5. Education was measured on a six-point scale, with scores ranging from low to high as: 1 = illiterate; 2 = private schooling or literacy class; 3 = primary school; 4 = junior high school; 5 = senior high school or vocational school; 6 = junior college and above.

### Statistical analyses

This research adopted descriptive statistical analyses and structure equation model (SEM) to analyze the data. Structural equation model analysis method has more advantages when dealing with the aggregate problems of measured variables and group comparison. Therefore, this paper uses SEM method to analyze the complex logical relationship between social network and the well-being of urban and rural elderly at different ages. In order to test whether the data fit the SEM analysis, we grouped all observed variables with 27 and 73 quartiles as critical values and performed a t-test. The results showed all variables are highly discriminative and are suitable for SEM analysis.

Multi-factor confirmatory analysis was performed on three measurement models of family network, friend network, and older adults’ well-being. The factor loading of all observation variables exceeds 0.5, and the reliability coefficient (SMC) is more than 0.25. All measurement models (CFA) have good reliability and validity, and are appropriate for SEM analysis.

The concept model was fitted based on the CLASS data. The output results show that the fitness indexes GFI and TLI failed to meet the standards, so the model needs to be optimized. The output of the model fitting shows that the value of correction index between the residua of “friends talking” and the residual of “relatives talking” is the largest. Therefore, after the correlation between the two is established, the model is re-fitted, and the result of GFI and TLI all meet the ideal standards. Meanwhile, RMSEA, X^2^, and DF have been further optimized, so the optimized model has a good fitness. (Table 2).

**Table 2:** Comparison of fit index before and after model optimization

	GFI	TLI	X^2^	DF	RMSEA
Pre-optimization model	0.890	0.859	1048	59	0.069
Optimized model	0.927	0.904	719	58	0.057
Ideal standard	> 0.9	> 0.9	–	–	< 0.08

## Results

### Descriptive statistics

The descriptive statistics of the main variables are shown in Table 3. From the mean of total sample of the social network, the mean values of the observed variables of the family network (family contacts, family talks, and family supports) are respectively 3.23, 2.26, and 3.02. Correspondingly, the mean values of the observed variables of the friend network (family contact, family talk, family support) are 2.58, 1.59, 1.79, respectively. Obviously, the mean values of family network are larger than those of the friend network. Through the comparison of urban and rural samples, it can be found that the difference in the mean of family network’ s observed variables is not obvious. However, regarding the mean of friend network’s observed variables, the values of rural sample are generally lower than those of urban sample. From the mean of total sample of the older adults’ well-being, life satisfaction is higher than self-rated health. Meanwhile, remarkably, life satisfaction and Self-rated health of rural elderly are both lower than that of urban elderly.

**Table 3:** Descriptive statistics

Latent variables		Observed variables	Mean of total sample	Mean of city sample			Mean of rural sample		
				The Young-old group	The Middle-old group	The Old-old group	The Young-old group	The Middle-old group	The Old-old group
Social network	Family network	Family contacts	3.23	3.25	3.29	3.29	3.12	3.18	3.36
		Family talks	2.26	2.29	2.29	2.31	2.20	2.24	2.10
		Family supports	3.02	2.94	3.04	3.18	2.95	3.10	3.21
	Friend network	Friend contacts	2.58	2.86	2.63	2.14	2.62	2.35	2.00
		Friend talks	1.59	1.84	1.60	1.37	1.55	1.43	1.17
		Friend supports	1.79	2.10	1.72	1.44	1.81	1.59	1.26
Older adults’ well-being		Life satisfaction	4.01	4.00	4.13	4.15	3.93	4.03	3.99
		Self-rated health	3.20	3.46	3.21	3.13	3.10	2.94	2.93
Control variables		Income	2.88	3.49	3.48	3.50	2.16	1.82	1.67
		Education	2.91	3.61	3.31	2.67	2.5	2.09	1.64
		Gender	0.52	0.50	0.55	0.56	0.48	0.54	0.58
		Age	70.31	63.76	74.35	83.84	63.78	74.20	83.58
		Number of children	3.09	2.10	3.28	3.93	2.83	4.13	4.88
		Marital status	0.35	0.20	0.40	0.64	0.22	0.48	0.76

In the control variables, the mean values of the total sample of income and education are 2.88 and 2.91, respectively. Moreover, the mean of each group of the urban sample is significantly higher than that of the rural. Regarding gender structure, 52% of the total sample are female and 48% are male. The gender ratio of the elderly in urban and rural areas is relatively balanced. The mean value of age’ total sample is 70.31, which is basically the same between urban and rural areas. The mean of the number of children in total sample is 3.09, that is, about three children per elderly Chinese family. The older the group samples are, the more children they have. The number of children in rural sample is significantly higher than that in urban. The mean value of the total sample for marital status is 0.35, meaning that 65% of the elderly are accompanied by a spouse, and the proportion of urban and rural elderly with spouses have gradually decreased with age.

### Structural characteristics: the urban-rural differences in age sequences in terms of older adults’ social networks and their well-being

**Fig. 1:**
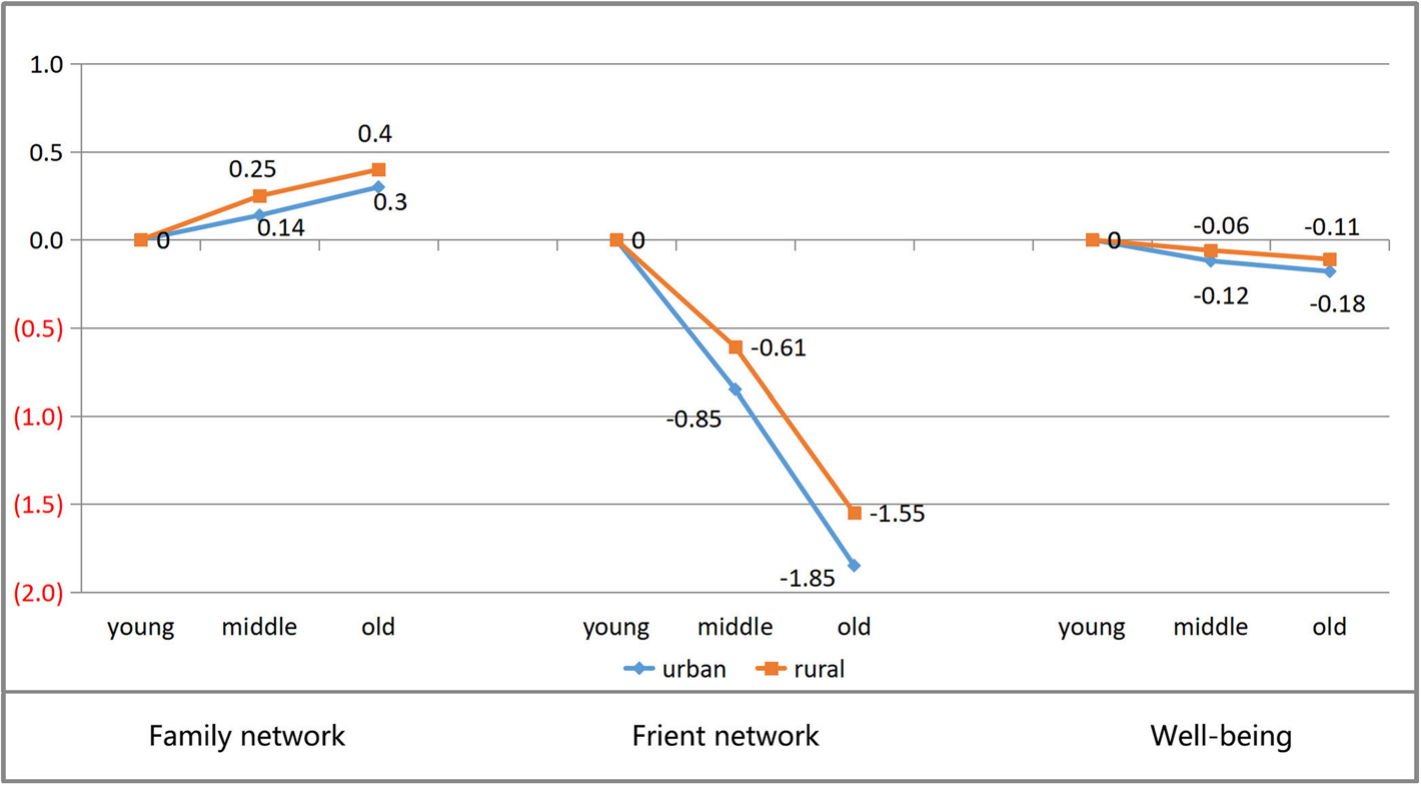
The comparison of family network, friend network and well-being between urban-rural elderly in different ages

We summed the latent variables of each group sample and compared them. Therefore, it can clearly reveal the changing trends of social network and older adults’ well-being at different ages in urban and rural China. Table 4 showed the mean values of latent variables, including family network, friend network and older adults’ well-being, at different age groups in urban and rural China. The statistical results of the difference comparison were shown in Fig. 1.

**Table 4:** The mean comparison of family network, friend network and well-being of the urban-rural elderly in different age groups

	Family network			Friend network			Older adults’ well-being		
	The young-old group	The middle-old group	The old-old group	The young-old group	The middle-old group	The old-old group	The young-old group	The middle-old group	The old-old group
**Urban**	8.48	8.62	8.78	6.8	5.95	4.95	7.46	7.34	7.28
**Rural**	8.27	8.52	8.67	5.98	5.37	4.43	7.03	6.97	6.92

With the increase of age, the gaps of urban and rural family network tend to be widened: for urban groups, the family network’s mean value of the middle-old is 0.14 more than that of the young-old, and the mean value of the old-old is 0.30 more than that of the young-old; for rural groups, the family network’s mean value of the middle-old is 0.25 more than that of the young-old, and that of the old-old is 0.40 more than that of the young-old. On the other hand, with the increase of age, the friend network of the elderly in urban and rural areas were gradually reduced: for urban groups, the average friend network’s mean value of the middle-old is 0.85 lower than that of the young-old, and that of the old-old is 1.85 lower than that of the young-old; for rural groups, the friend network’s mean value of the middle-old is 0.61 lower than that of the young-old, and the mean value of the old-old is 1.55 lower than that of the young-old.

Meanwhile, the older adults’ well-being in urban and rural areas dropped slightly as they grow older. For urban groups, the mean value of well-being of the middle-old is 0.12 lower than that of the young-old, and the old-old is 0.18 lower than that of the young-old. In comparison, the decline in well-being of rural elderly is smaller than that of urban elderly: The mean of the middle-old is only 0.06 lower than that of the young-old, and the mean of the old-old is only 0.11 lower than that of young-old.

### Mechanism of well-being efficacy: an urban-rural comparison of how social network influence well-being of the elderly at different ages

**Fig. 2:**
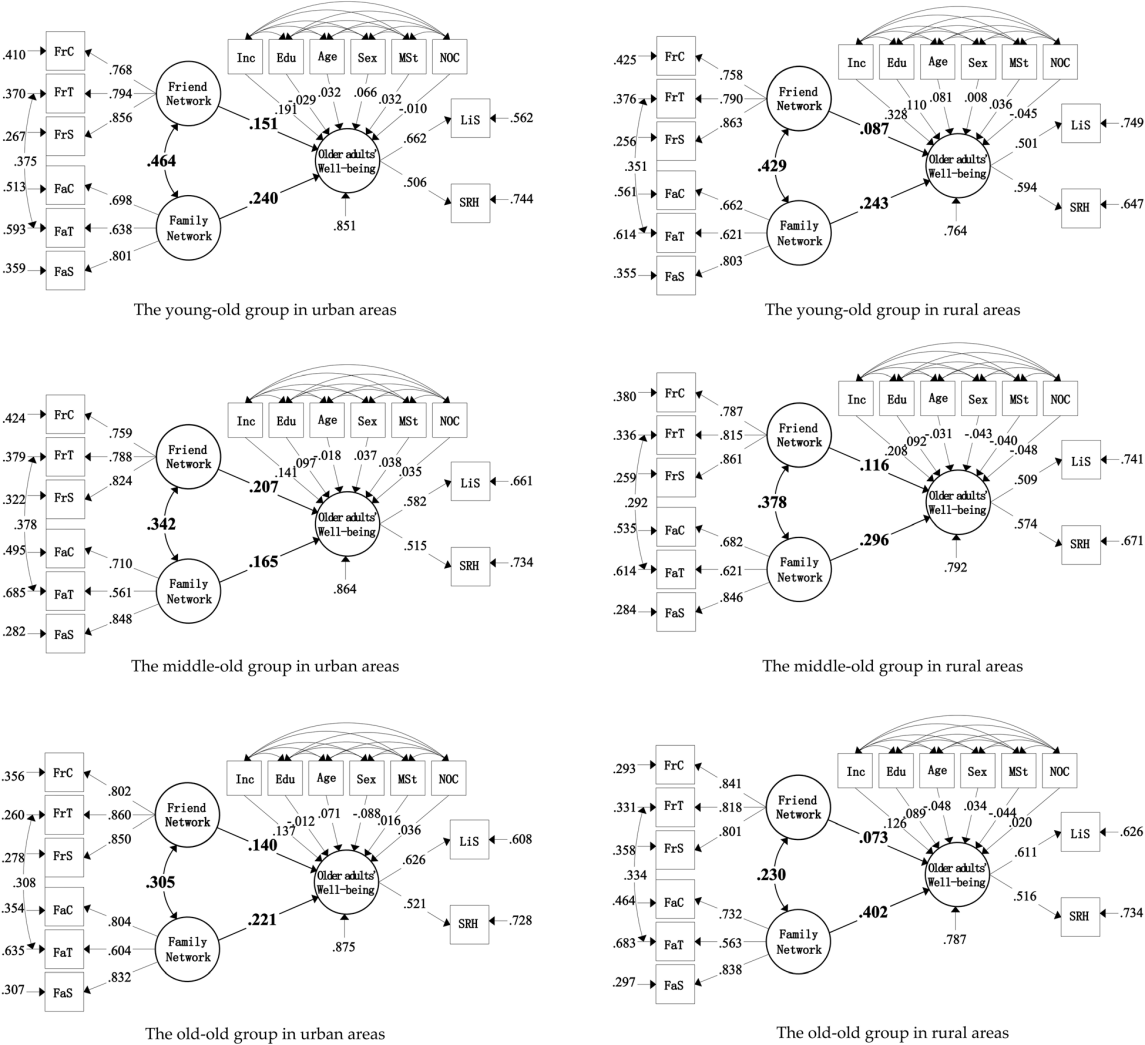
The comparison of standardized path of urban and rural age models

We have made groups comparison of the models in three age stage groups of urban-rural elderly, separately. The results showed: *P* value of the rural group comparison is 0.026 < 0.05; *p* value of the urban group comparison is 0.082 > 0.05. These results indicated that there is significant difference in rural group, but not in urban group. The comparison of group model fitting results was shown in Table 5, and the standardized path was shown in Fig. 2.

**Table 5:** An urban-rural comparison of group model fitting results at different age groups

Independent variables		Dependent variables: older adults’ well-being					
		Rural elderly			Urban elderly		
		The young-old group	The middle-old group	The old-old group	The young-old group	The middle-old group	The old-old group
Independent variables: Social network	Family network	0.243^a^	0.296^a^	0.402^a^	0.240^a^	0.165^a^	0.221^a^
	Friend network	0.087^b^	0.116^b^	0.073	0.151^a^	0.207^a^	0.140^a^

Notes: ^a^ means significance at the 0.01 confidence level; ^b^ means significance at the 0.05 confidence level

There were obvious differences between the influence of family network and friend network on the older adults’ well-being. First, family network greatly enhance the well-being of all urban and rural elderly groups. From younger to older groups, the total effect values of urban elderly’s well-being affected by family network are 0.240 (*P* = 0.000), 0. 165 (*P* = 0.001) and 0. 221**(*P* = 0.001). Correspondingly, from younger to older groups, the figures of the three age groups of rural elderly are respectively 0.243(*P* = 0.000), 0.296(*P* = 0.000) and 0.402(*P* = 0.000). The effect of family network on the rural elderly’s well-being shows a remarkable increase with age. However, the impact on the urban elderly of different age groups has not changed significantly.

Second, the influence of friend network on older adults’ well-being varies significantly between urban and rural groups. Friend network have a positive effect for all urban elderly. With increasing age, the effect values are 0.151 (*P* = 0.000), 0.207(*P* = 0.000), 0.140(*P* = 0.007), respectively. On the other hand, for rural elderly, friend network can only influence the well-being of the young-old and middle-old groups with the effect value of 0.087(*P* = 0.002) and 0.116(*P* = 0.012), respectively. Its impact on the well-being of the old-old group has no significant effect.

## Discussion

Our study has compared older adults in China’s urban and rural areas, and found that the social network and its efficacies on well-being between urban-rural elderly have both shown great differences.

Our research confirms the conclusion of some scholars that there are significant differences in the characteristics of old adult’ social network among different age groups [25–28]. In addition, interestingly, we have found some peculiar characteristics of the old adult’ social network in China. First, with the increase of age, the structure of the old adults’ social network shows a reverse progressive sequence. That is, as the elderly grow old, their family network expand while their friend network shrink gradually. We call this characteristic of the old adults’ social network as the “reverse structure “ in age sequences. Secondly, there are differences between urban and rural elderly in the characteristic of “reverse structure”, which are reflected in the two dimensions of family network and friend network. Specifically, the expansion scale of family network of rural elderly with age is significantly greater than that of urban. Correspondingly, the reduction of friend network of rural elderly is smaller than that of urban elderly. Thirdly, the well-being of the elderly shows a decreasing trend with age. However, there is a slight difference between urban and rural areas, that is, the decline trend of well-being of rural group is smaller than that of urban group.

We believe that the “reverse structure” characteristic of Chinese elderly’s social network can be explained from subjective and objective perspectives. The subjective reason is that as the age increases, the physiological functions of the elderly gradually decline, and more emotional and material support in life are expected from their families. The objective reason is that the fertility status of the older elderly group is not affected by the family planning policy, whose impact on the young-old and the middle-old groups is growing, instead. Inevitably, the number of children from older to younger senior citizens has dropped accordingly, and the family size are bound to shrink in the end.

Our research validated the conclusion that different dimensions of social network have different effects on the older adults’ well-being [8, 22, 23]. The view that social network have significant effects on the older adults’ well-being at different ages has only been confirmed in the rural group [25–28], but not in the urban group. For the rural elderly, we found that the family network was more important to their well-being than the friend network. Specifically, the family network has a significant influence on the well-being of rural elderly in all age groups, and it gradually increases with age. However, the friend network has a significant influence lightly only on the young-old and middle-old age groups. This conclusion is contrary to the findings of some scholars based on Western elderly samples [17, 24]. They found that the influence of friend network on the well-being of the elderly was more important than that of family network.

Compared with the rural elderly, the family network has a smaller efficacicy on the well-being of the urban elderly. In fact, for the urban elderly, the friend network and the family network have an equally important efficiency on their well-being. This is a very interesting finding. We believe that the important reason for the different trends in the well-being of urban and rural elderly is the difference in the impact of social network on their well-being.

With the increase of age, the physical functions of older adults gradually decline and they even have to face death. But for the rural elderly, the trend of their well-being decline with age is not great. This is because the support from their family network greatly compensates their sense of loss when aging. The family network scale of the rural elderly grows accordingly with age. Meanwhile, family network’s efficacies on their well-being are correspondingly increasing. This compensatory support from the family network which grows with age allows progressive enhancement of the well-being of the middle-old and the old-old groups in rural areas. This is the crucial reason that explains why the well-being of rural elderly doesn’t decline dramatically with age.

On the other hand, family network had certain positive efficacies on the well-being of urban elderly. However, compared with the rural elderly, family network of the urban elderly was much smaller in terms of both expansion scale and its impact on the well-being. As a result, the compensatory efficacy of family network on the well-being of the urban elderly is much smaller. For another, although the friend network played an important role in the well-being of the urban elderly, with the growth of age, their friend network has been greatly shrunk. Due to this negative impact from the shrinking friend network plus the insufficient compensatory efficacy of family network, the decline trend of the urban elderly well-being with age was greater than that of the rural elderly.

It needs to be addressed that limitations still exist in this research. First, all statistics quoted are from the 2014 China Longitudinal Aging Social Survey (CLASS) while some social changes have taken place afterwards and so have the elderly groups in different age. Therefore, future discussions should be based on more up-to-date statistical information. Secondly, this longitudinal survey conducted the measurement of subjects’ social network from two dimensions: family network and friend network. In fact, more detailed dimensions may facilitate in-depth discussions on the issue of old adults’ social network, such as adding neighbor network etc. Thirdly, older adults’ well-being in this article is based on subjective assessments, i.e., how people feel. Further research regarding social network efficacies on old adults’ well-being needs to be done by using objective measurements data.

## Conclusion

As a key to “active aging”, the efficacies of social network lie not only in effectively improving older adults’ well-being and their life quality. They also positively contribute to the old-age security improvement, alleviating the problems of elderly care in China and promoting the healthy development of the entire country. Based on the large sample of data (CLASS), we found that the social network characteristics of the Chinese elderly are different between different age stages. Namely, the family network and the friend network have the “reverse structure “ in age sequences. Furthermore, there are obvious urban-rural differences in the impact of social network on the well-being of the Chinese elderly. For the rural elderly, the family network has a strong positive effect on their well-being than the friend network, and the intensity of this effect gradually increases with age. However, for the urban elderly, the impact of family network on their well-being is just as important as that of the friend network.

Considering the differences in the efficacies of social network on the well-being of the elderly in urban and rural areas, it is essential to adopt separate approaches when trying to enhance older adults’ well-being in China. To improve the well-being of the rural elderly, attention should be paid to expanding the size of their family network, which can be achieved by expanding the size of the family, encouraging the strengthening of the ties between relatives, etc. Family scale is the basis of family network scale. In current China, with social changes and development, the culture of filial piety and birth rate are naturally weakening or declining. Therefore, we appeal for full liberalization of fertility restrictions, promotion of incentive fertility policies, reduction of economic pressure on youth groups, and improvement of the education system. It is expected that the comprehensive effect in many aspects can bring about the improvement of fertility rate, and then increase the scale of the family. To improve the well-being of the urban elderly, it is equally important to expand scale of their family network and friend network. Therefore, apart from the above-mentioned measures, it is also necessary to pay attention to construct and optimize the environment for socializing, and actively create and cultivate a good atmosphere of social interaction, in order to expand the scale of the friend network of the elderly.

## Data Availability

The population data (CLASS) that support the findings of this study are available from http://class.ruc.edu.cn/

## Abbreviations

SEM: Structural equation modeling
CLASS: China longitudinal aging social survey
PSU: Primary sampling units
SSU: Secondary sampling units
CHY: Chinese yuan
SMC: Squared multiple correlation
CFA: Confirmatory factor analysis
GFI: Goodness-of-fit index
TLI: Tucker-Lewis index
RMSEA: Root mean square error of approximation
DF: Degree of freedom
